# The Unusual Increase in Suicides Among Women in Japan During the COVID-19 Pandemic: A Time-series Analysis Until October 2021

**DOI:** 10.2188/jea.JE20220186

**Published:** 2023-01-05

**Authors:** Kohtaro Kikuchi, Tatsuhiko Anzai, Kunihiko Takahashi

**Affiliations:** 1 Department of Biostatistics, M&D Data Science Center, Tokyo Medical and Dental University, Tokyo, Japan; 2 Japanese Red Cross Musashino Hospital, Tokyo, Japan

**Keywords:** suicide, COVID-19 pandemic, time-series approach

## Abstract

**Background:**

Japan has witnessed an unusual increase in the number of suicides among women during the coronavirus disease 2019 pandemic. An analysis is required to identify the influencing factors during the pandemic and develop new measures for preventing suicides.

**Methods:**

Data on the number of monthly suicides were collected from the National Police Agency of Japan. The expected number of suicides among women during the pandemic was estimated using a time-series model based on pre-pandemic data, considering year-to-year trends. The observed-to-expected (O/E) ratio of suicides was estimated from March 2020 to October 2021 using job status, suicide motive, and age.

**Results:**

The number of suicides among women in Japan increased beyond the expected number until October 2021. The O/E ratio based on job status, suicide motive, and age (except self-employed, unknown job status, and women aged ≥80 years) was significantly above 1.0 from March–December 2020, and the increase in suicides continued in almost all categories in 2021.

**Conclusion:**

Although several reasons were reported for increased suicides among women in Japan during the pandemic (eg, economic downturn, financial instability, and loneliness), suicides increased irrespective of job status, suicide motive, or age. Comprehensive measures to prevent suicide might have been important during the pandemic, instead of limiting interventions to the reported specific population.

## INTRODUCTION

Despite substantial mitigation efforts, the suicide rate in Japan has been alarming among both men and women.^1^^,^^2^ Japan saw an unusual rise in suicides among women during the coronavirus disease 2019 (COVID-19) pandemic. The increase was 37% higher than the pre-pandemic period, particularly between July and October 2020.^2^^–^^4^ Surprisingly, such an increase has not been observed in other developing countries.^5^^,^^6^ This unusual increase in women’s suicides during the pandemic is a serious concern, particularly as the COVID-19-related mortality rate is declining with widespread vaccination.^7^

Before the COVID-19 pandemic, Japan had the second highest suicide rate among women globally, with 8.6 suicides per 100,000 women.^8^ The stress from personal responsibilities, such as family obligations,^9^^,^^10^ was considered a key cause impacting this high prevalence. During the pandemic, the economic downturn, worsening unemployment, and exacerbation of psychiatric disorders—more frequent among women than men—could have contributed to the increased numbers in psychologically vulnerable Japanese women.^11^^,^^12^ We need to identify the factors influencing suicide among women during the pandemic to prevent suicide as the pandemic continues. Previous studies have reported increased suicide rates for multiple women’s categories.^13^^,^^14^

These numbers of suicides have been associated with annual changes in the unemployment rate; the trends change depending on such factors.^15^^,^^16^ However, previous studies investigating categories^13^^,^^14^ have ignored these trends. This may have led to the underestimation of their impact on suicides during this pandemic.

To address this research gap, we evaluated the observed-to-expected (O/E) ratio of female suicides in Japan from March 2020 to October 2021, which were categorized using job status, suicide motive, and age. A time-series trend analysis was considered to assess the suicide and unemployment rate before the pandemic. The O/E subgroup ratios based on the COVID-19 infection was compared to evaluate regional differences.

## METHODS

### Data

We downloaded the monthly number of female suicides from January 2013 to October 2021 collected by the National Police Agency from the website of the Ministry of Health, Labour and Welfare.^13^ March–December 2020 was defined as Term I and January–October 2021 as Term II; emergency declarations and steps to prevent infection spread continued intermittently in Term II.

The monthly female suicides were categorized by job status, suicide motive, and age. The job status categories were as follows: self-employed, employee, student, housewife, unemployed, pension/unemployment insurance liver, other non-worker, and unknown. Suicide motives included family, health, economic, work-related, relationship, school-related, other problems, and unknown reasons; each suicide had one to three motives. The ages of the women who attempted suicide were categorized as: <20, 20–29, 30–39, 40–49, 50–59, 60–69, 70–79, and ≥80 years. Eleven suicides in the “unknown” age category were excluded.

Monthly provisional female population^17^ and unemployment rates^18^ were obtained from the Statistics Bureau of Japan. The prefecture population was allocated using each year’s composition ratio. The cumulative number of COVID-19 cases in the prefectures for January 2021 were collected using a web-based system.^19^

A formal ethical review was not required as public aggregate data were used.

### Model

The O/E ratio was defined as the total number of observed women’s suicides in Japan divided by the total number of expected suicides during a non-pandemic period. The O/E ratio for each category was derived as:

$$\frac{\Sigma_{t}O_{t}}{\Sigma_{t}E_{t}}$$

where *O_t_* and *E_t_* represented the observed and expected number of suicides during the month *t* (*t* = 1,…,*T*) in Terms I and II, respectively.

We used the following time-series model to estimate *E_t_* in terms of the number of suicides (*y_t_*) based on female suicide trends in each category from January 2013 to February 2020^3^:

$$y_{t}∼\text{Poisson}(\mu_{t})$$

$$\begin{array}{rl} \log(\mu_{t}) & =\beta_{0}+\sum_{M=1}^{12}\beta_{M}I({\text{month}}_{t}=M)\\ & \;+\beta_{U1}u_{t}+\beta_{U2}u_{t-1}+\log({\text{pop}}_{t}) \end{array}$$

where *month_t_* represented the month during *t* and *I*(·) is the indicator function. *u_t_* and *u_t_*_−1_ represented the unemployment rates during *t*, and *t* − 1, and *pop_t_* represented the population during *t*.

The coefficients—*β*_0_, *β_M_* (*M* = 1, 2,…,12), *β_U_*_1_ and *β_U_*_2_—were estimated in the model where *β_M_* at *t* is set to zero for *M* = 1. Although in a previous study, the time-series model included the year,^16^ we excluded it from our model due to the strong correlation between the unemployment rate and time.

Based on our estimated model, we calculated *E_t_* for Terms I and II. We substituted *u_t_* with the mean unemployment rate at *t* in 2018 and 2019 to evaluate the pandemic’s impact, as the unemployment rate had been stable in the beginning of 2018 until the COVID-19 outbreak.^18^

### Statistical analysis

First, we plotted the number of women’s suicides between January 2013 and October 2021 using a 95% prediction interval (PI). Our analysis focused on data before February 2020, and we applied a model proposed in our study^16^ that included the year effect. We also plotted the number of men’s suicides.

Next, we estimated the O/E ratios for the categories of job status, suicide motive, and age for Terms I and II, provided the profile-likelihood-based 95% confidence interval (CI) of the O/E ratio and its forest plots, and conducted sensitivity analyses on the unemployment rates in 2018 and 2019.

To evaluate regional difference due to COVID-19 infection, we estimated O/E ratios from March 2020 to October 2021 in subgroups defined as cumulative number of cased <1,000, 1,000 to 2,000 and ≥2,000 per million population.

The significance level was set at *P* < 0.05 for all statistical analyses, which were conducted using the R software (version 4.1.3; R Foundation for Statistical Computing, Vienna, Austria).

## RESULTS

**Figure 1.**
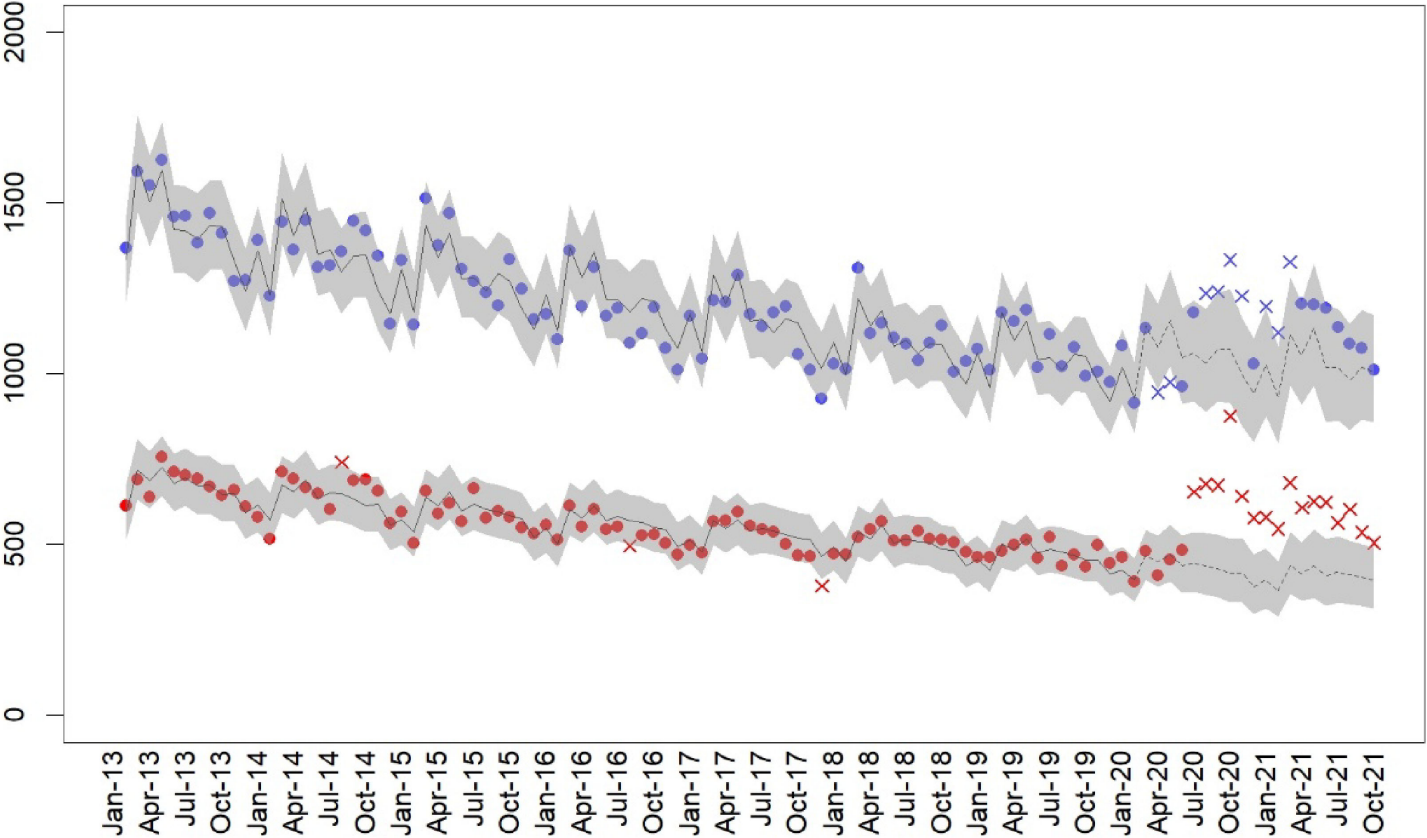
Number of suicides among females (red) and males (blue) from January 2013 to October 2021. The dots represent the observed number of suicides within the 95% prediction interval (marked by the gray strip); The “x” represents the observed number of suicides outside the 95% prediction intervals. The solid line marks the predicted number of suicides in non-pandemic periods, whereas the dashed line represents the predicted number of suicides during the pandemic.

The number of women’s suicides during the pandemic period after March 2020 was 11,786. Since July 2020, the number of women’s suicides across all months have increased beyond the interval predicted by the steadily decreasing suicide trend since 2013, the non-pandemic period (Figure 1). Although suicides among men have also been increasing, the increase is greater among women.

**Figure 2.**
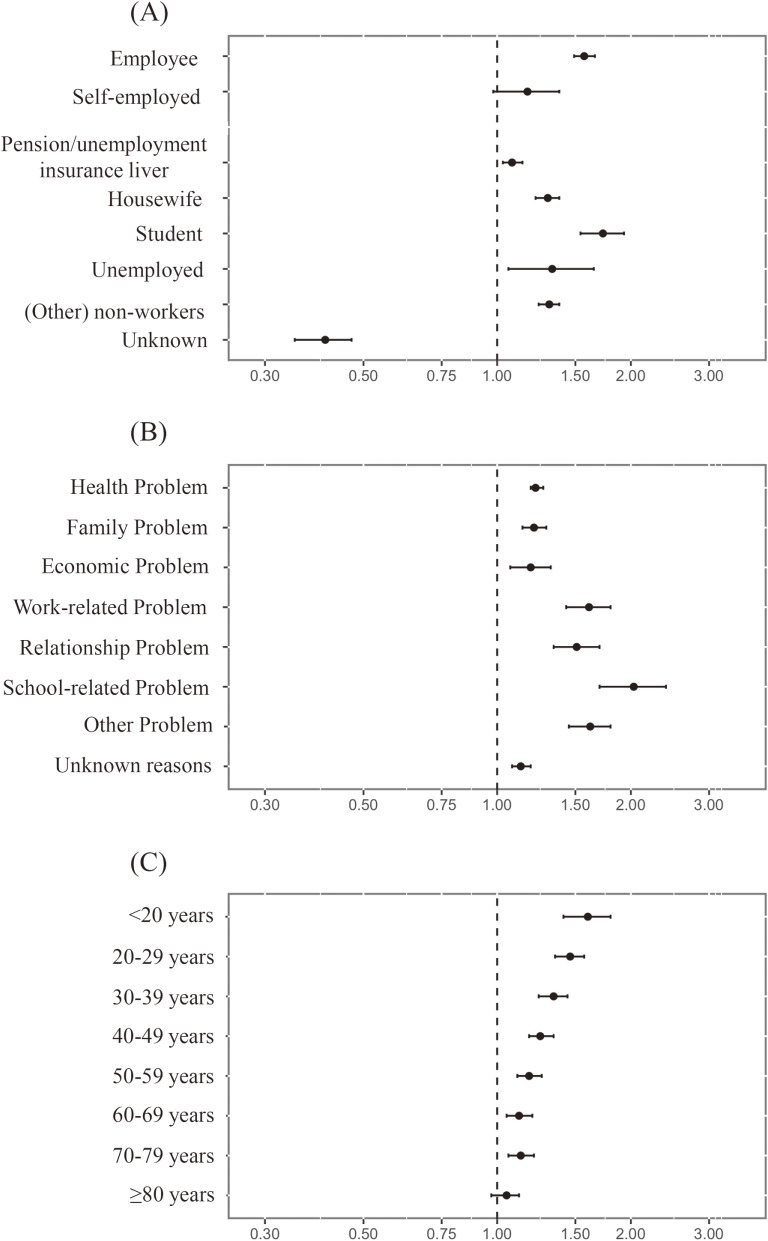
Forest plot of O/E ratio by (**A**) job status, (**B**) motive, and (**C**) age during Term I (March to December, 2020). O/E ratio, ratio of observed to expected results.

The categorical O/E ratios increased for all job statuses except self-employment during Term I, compared to the expected number based on the pre-pandemic trend. The O/E ratios for these job statuses were statistically higher than 1.0 (Table 1). The highest O/E ratio among job categories was observed for “student” (O/E ratio 1.73; 95% CI, 1.54–1.93). The O/E ratios for employees and non-workers, except the category “Pension and unemployment insurance liver,” were greater than 1.30. By contrast, the observed number in the “unknown” category was lesser than the expected (O/E ratio 0.41; 95% CI, 0.35–0.47). For the motives category, all the O/E ratios were significantly greater than 1.0 (Table 1), with the highest being 2.03 (95% CI, 1.70–2.40) owing to a “school-related problem” and the lowest being 1.13 (95% CI, 1.08–1.19) owing to “unknown reasons.” Similarly, the O/E ratio for women aged “0–19 years” was higher than 1.0, and the point estimate was 1.60 (95% CI, 1.41–1.80). The O/E ratios were negatively associated with age (Figure 2).

**Table 1.** Estimated O/E ratio and its 95% confidence interval per job status, motive, and age category during Term I (March to December, 2020)

	Total number of observations (Σ*_t_O_t_*)	Total number of expectations (Σ*_t_E_t_*)	O/E ratio (95% CI)	*P*-value
**Job status**				
Worker				
Employee	1,281	813.4	1.57 (1.49–1.66)	<0.001
Self-employed	136	116.4	1.17 (0.98–1.38)	0.069
Non-worker				
Pension and unemployment insurance liver	1,623	1,495.9	1.08 (1.03–1.14)	0.001
Housewife	989	763.1	1.30 (1.22–1.38)	<0.001
Student	304	176.0	1.73 (1.54–1.93)	<0.001
Unemployed	78	58.6	1.33 (1.06–1.65)	0.012
Other non-worker	1,323	1,008.1	1.31 (1.24–1.38)	<0.001
Unknown	192	473.8	0.41 (0.35–0.47)	<0.001

**Motive**				
Health	3,745	3,049.6	1.22 (1.19–1.27)	<0.001
Family	1,052	867.3	1.21 (1.14–1.29)	<0.001
Economic	337	283.0	1.19 (1.07–1.32)	0.001
Work-related	278	172.9	1.61 (1.43–1.80)	<0.001
Relationship	268	177.3	1.51 (1.34–1.70)	<0.001
School-related	129	63.5	2.03 (1.70–2.40)	<0.001
Other	341	210.8	1.62 (1.45–1.80)	<0.001
Unknown reasons	1,530	1,348.9	1.13 (1.08–1.19)	<0.001

**Age, years**				
<20	260	162.7	1.60 (1.41–1.80)	<0.001
20–29	695	477.1	1.46 (1.35–1.57)	<0.001
30–39	657	491.1	1.34 (1.24–1.44)	<0.001
40–49	925	737.4	1.25 (1.18–1.34)	<0.001
50–59	892	754.6	1.18 (1.11–1.26)	<0.001
60–69	798	710.6	1.12 (1.05–1.20)	0.001
70–79	930	822.1	1.13 (1.06–1.21)	<0.001
≥80	762	729.1	1.05 (0.97–1.12)	0.223

CI, confidence interval; O/E ratio, ratio of observed to expected results.

**Figure 3.**
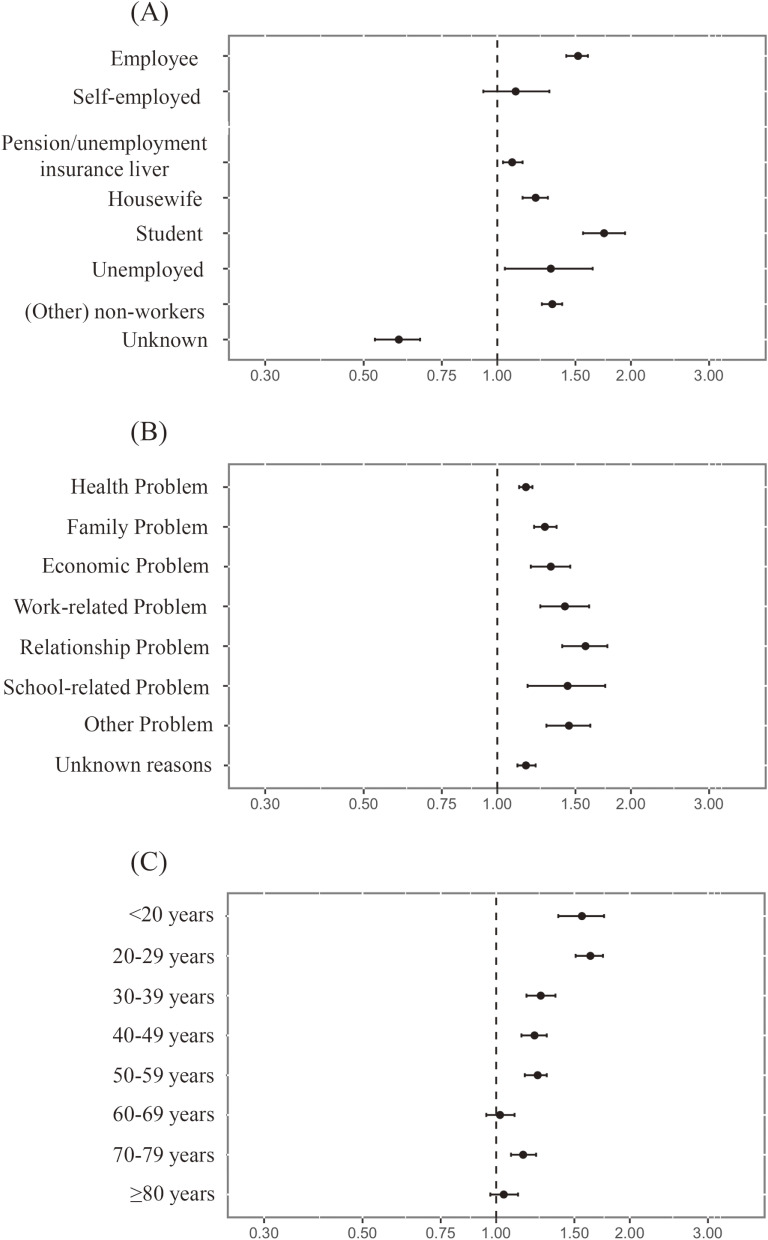
Forest plot of O/E ratio by (**A**) job status, (**B**) motive, and (**C**) age during Term II (January to October, 2021). O/E ratio, ratio of observed to expected results.

Even in Term II, the O/E ratios statistically exceeded 1.0 in several categories (Table 2 and Figure 3). The O/E ratio for women aged “60–69 years” was not statistically significant. For other categories, the direction and detection of significant differences were same for both Terms I and II.

**Table 2.** Estimated O/E ratio and its 95% confidence interval per job status, motive, and age category during Term II (January to October 2021)

	Total number of observations (Σ*_t_O_t_*)	Total number of expectations (Σ*_t_E_t_*)	O/E ratio (95% CI)	*P*-value
**Job status**				
Worker				
Employee	1,227	809.8	1.52 (1.43–1.60)	<0.001
Self-employed	129	116.5	1.10 (0.93–1.31)	0.249
Non-worker				
Pension and unemployment insurance liver	1,612	1,487.9	1.08 (1.03–1.14)	0.001
Housewife	908	745.5	1.22 (1.14–1.30)	<0.001
Student	323	185.5	1.74 (1.56–1.94)	<0.001
Unemployed	73	55.4	1.32 (1.04–1.64)	0.019
Other non-worker	1,303	980.5	1.33 (1.26–1.40)	<0.001
Unknown	293	489.9	0.60 (0.53–0.67)	<0.001

**Motive**				
Health	3,483	3,010.6	1.16 (1.12–1.20)	<0.001
Family	1,096	854.8	1.28 (1.21–1.36)	<0.001
Economic	370	280.5	1.32 (1.19–1.46)	<0.001
Work-related	244	171.7	1.42 (1.25–1.61)	<0.001
Relationship	277	175.6	1.58 (1.40–1.77)	<0.001
School-related	93	64.7	1.44 (1.17–1.75)	<0.001
Other	301	207.9	1.45 (1.29–1.62)	<0.001
Unknown reasons	1,564	1,345.8	1.16 (1.11–1.22)	<0.001

**Age, years**				
<20	264	169.7	1.56 (1.38–1.75)	<0.001
20–29	771	473.9	1.63 (1.51–1.74)	<0.001
30–39	623	493.6	1.26 (1.17–1.36)	<0.001
40–49	896	732.6	1.22 (1.14–1.30)	<0.001
50–59	910	732.6	1.24 (1.16–1.32)	<0.001
60–69	722	707.0	1.02 (0.95–1.10)	0.572
70–79	936	813.3	1.15 (1.08–1.23)	<0.001
≥80	745	716.1	1.04 (0.97–1.12)	0.285

CI, confidence interval; O/E ratio, ratio of observed to expected results.

There was no difference in order and directions in the O/E ratios for various categories between the main analysis and the sensitivity analysis (eTable 1 and eTable 2). The subgroup O/E ratios were approximately ≥1.0 for most categories (Table 3), with tend to be higher in prefectures with more infected cases.

**Table 3.** Estimated O/E ratio and its 95% confidence interval by categories and subgroups March, 2020 to October, 2021

	Prefecture subgroup					

	<1,000 cases (per million population) (31 prefectures)		1,000–2,000 cases (per million population) (10 prefectures)		≥2,000 cases (per million population) (6 prefectures)	

	Total number of observations/ expectations	O/E ratio (95% CI)	Total number of observations/ expectations	O/E ratio (95% CI)	Total number of observations/ expectations	O/E ratio (95% CI)
**Job status**						
Worker						
Employee	798/589.1	1.35 (1.26–1.45)	746/505.9	1.47 (1.37–1.58)	1,010/484.9	2.08 (1.96–2.21)
Self-employed	105/96.4	1.09 (0.89–1.31)	67/62.4	1.07 (0.84–1.35)	93/63.8	1.46 (1.18–1.78)
Non-worker						
Pension and unemployment ​ insurance liver	1,178/1,062.8	1.11 (1.05–1.17)	1,088/987.6	1.10 (1.04–1.17)	989/872.1	1.13 (1.06–1.21)
Housewife	618/535.8	1.15 (1.06–1.25)	590/474.6	1.24 (1.15–1.35)	705/468.1	1.51 (1.40–1.62)
Student	181/123.8	1.46 (1.26–1.69)	200/136.8	1.46 (1.27–1.67)	264/93.3	2.83 (2.50–3.19)
Unemployed	59/49.5	1.19 (0.91–1.52)	38/27.7	1.37 (0.98–1.86)	56/33.6	1.67 (1.27–2.14)
Other non-worker	858/686.1	1.25 (1.17–1.34)	712/649.1	1.10 (1.02–1.18)	1,124/575.0	1.95 (1.84–2.07)
Unknown	21/13.5	1.56 (0.98–2.32)	21/17.8	1.18 (0.74–1.76)	39/910.8	0.04 (0.03–0.06)
**Motive**						
Health	2,248/1,983.5	1.13 (1.09–1.18)	2,556/2,081.6	1.23 (1.18–1.28)	2,505/1,807.1	1.39 (1.33–1.44)
Family	726/606.8	1.20 (1.11–1.29)	641/497.3	1.29 (1.19–1.39)	778/547.6	1.42 (1.32–1.52)
Economic	224/191.6	1.17 (1.02–1.33)	204/178.8	1.14 (0.99–1.30)	282/172.8	1.63 (1.45–1.83)
Work-related	171/123.3	1.39 (1.19–1.61)	149/105.5	1.41 (1.20–1.65)	211/103.3	2.04 (1.78–2.33)
Relationship	166/119.2	1.39 (1.19–1.62)	155/106.5	1.46 (1.24–1.70)	235/118.5	1.98 (1.74–2.25)
School-related	70/42.1	1.66 (1.30–2.08)	70/51.4	1.36 (1.07–1.71)	84/31.2	2.69 (2.16–3.31)
Other	227/162.9	1.39 (1.22–1.58)	192/108.6	1.77 (1.53–2.03)	232/129.0	1.80 (1.58–2.04)
Unknown reasons	1,097/791.1	1.39 (1.31–1.47)	474/421.6	1.12 (1.03–1.23)	1,189/1,392.9	0.85 (0.81–0.90)
**Age, years**						
<20	176/110.1	1.60 (1.37–1.85)	154/109.5	1.41 (1.20–1.64)	198/110.4	1.79 (1.55–2.05)
20–29	414/278.4	1.49 (1.35–1.63)	401/269.6	1.49 (1.35–1.64)	636/395.8	1.61 (1.49–1.73)
30–39	398/290.6	1.37 (1.24–1.51)	377/285.5	1.32 (1.19–1.46)	493/405.7	1.22 (1.11–1.33)
40–49	574/419.2	1.37 (1.26–1.48)	570/445.8	1.28 (1.18–1.39)	663/598.3	1.11 (1.03–1.19)
50–59	553/467.9	1.18 (1.09–1.28)	548/439.1	1.25 (1.15–1.36)	698/576.2	1.21 (1.12–1.30)
60–69	517/539.7	0.96 (0.88–1.04)	469/427.6	1.10 (1.00–1.20)	520/445.8	1.17 (1.07–1.27)
70–79	662/616.6	1.07 (0.99–1.16)	556/494.1	1.13 (1.03–1.22)	644/519.9	1.24 (1.15–1.34)
≥80	688/644.3	1.07 (0.99–1.15)	390/386.5	1.01 (0.91–1.11)	432/411.2	1.05 (0.95–1.15)

CI, confidence interval; O/E ratio, ratio of observed to expected results.

Range of cumulative number of infected COVID-19 case per million population was 148 to 4,389 at January 2021.

## DISCUSSION

The global suicide rate declined during the COVID-19 pandemic’s first stage.^20^ Although the number initially declined in Japan,^16^ it changed by July 2020; the numbers began increasing, particularly among women.^3^^,^^4^ Previous studies have suspected specific causes for the increase in suicides among women during the pandemic in Japan, such as economic downturn and financial instability.^11^^,^^21^ However, our study reveals that the number of suicides increased irrespective of job status, suicide motive, or age. The implication is that the pandemic created an additional burden irrespective of categories. For all categories of women, no significant increase in suicides exceeded the upper bound of the 95% PI before June 2020, as indicated by Anzai et al,^16^ was confirmed, followed by an increase (eFigure 1, eFigure 2, and eFigure 3).^3^^,^^4^

The O/E ratios for categories characteristic of most young women (ie, student, unemployed, school-related suicide motive) were relatively higher than those in other categories. A recent report showed that young women are susceptible to COVID-19 fears and the stigma associated with it.^22^ Another report stated that the increasing number of suicides among female students is a result of psychological problems, such as depression, career anxiety, and academic slumps.^2^ Suicidal ideation has been reported to prevail among women and young people; thus, monitoring this trend is important.^6^

Suicidal behaviors are highly complex and based on multiple dimensions.^5^ Numerous common factors might be affecting the suicide rates in Japan during the pandemic. For example, due to shutting down of schools, daycare centers, and inaccessible formal medical care, Japanese women have to personally care for family members, owing to gender stereotypes in the Japanese culture,^23^^,^^24^ thus leading to poor mental health and suicide.^25^^,^^26^ Including common factors—such as official financial support by the government^27^ and specific aspects based on the investigated categories—is important to prevent suicide among Japanese women during the pandemic.

The unemployment rate should be considered as an indicator reflecting social conditions that positively correlates with suicide in Japanese women. Thus, the unemployment rate, specifically that of the previous month, influences suicides in women—irrespective of job status, suicide motive, and age category.^15^ This factor only negatively influenced the numbers of unknown job status. Although there are several suicide-related factors, most observations were included in the PI throughout the non-pandemic period. Deviations of this model were smaller for all categories compared to the model without the unemployment rate. Thus, we could provide a reasonable expected number under “normal” conditions (eFigure 1, eFigure 2, and eFigure 3).^16^

The expected values were calculated based on the unemployment rates of 2018 to 2019, continuing to the pandemic period, along with the O/E ratios. Our method could remove the bias of ignoring the unemployment rate while comparing suicide numbers, and the bias from using predicted data-based numbers that include the pandemic impact.^13^^,^^14^ Consequently, the results revealed that the unusual increase in female suicides has yet to decline in Term II for most categories. Although the increase is not limited to certain prefectures based on infected cases’ frequency, the increase might be related to restricted activity or anxiety due to widespread infection. Note that the results for the unknown category of job status might be affected by changes in survey method and other factors. Future work to investigate the factors influencing such regional differences might be important.

This study has some limitations. First, data were collected on three suicide motives; however, a combination of the three could not be evaluated. There is potential bias, since no scientific measures can accurately determine motives. Second, we could not determine how COVID-19 affected suicides among women in Japan. However, as the data were collected over an extended period, a meaningful analysis of the difference between the observed and expected numbers was possible. Third, although we only analyzed the suicidal trends among women until October 2021, both the pandemic and trend of increased suicides are ongoing. While further long-term research important, we should consider the changed relationship between suicide and unemployment rate in the post-pandemic period. Another future direction could be studying the timing of the suicide attempts during the pandemic using questionnaires, possibly highlighting unforeseen critical issues.

## SUPPLEMENTARY MATERIAL

eTable 1. Estimated O/E ratio and its 95% confidence interval per job status, motive, and age category during Term I (March to December, 2020) as sensitivity analysis

eTable 2. Estimated O/E ratio and its 95% confidence interval per job status, motive, and age category during Term II (January to October, 2021) as sensitivity analysis

eFigure 1. Number of suicides among females by job status categories from January 2013 to October 2021. The dots represent the observed number of suicides within the 95% prediction interval (marked by the gray strip); The “x” represents the observed number of suicides outside the 95% prediction intervals. The solid line marks the predicted number of suicides in non-pandemic periods, whereas the dashed line represents the predicted number of suicides during the pandemic.

eFigure 2. Number of suicides among females by motive categories from January 2013 to October 2021. The dots represent the observed number of suicides within the 95% prediction interval (marked by the gray strip); The “x” represents the observed number of suicides outside the 95% prediction intervals. The solid line marks the predicted number of suicides in non-pandemic periods, whereas the dashed line represents the predicted number of suicides during the pandemic.

eFigure 3. Number of suicides among females by age categories from January 2013 to October 2021. The dots represent the observed number of suicides within the 95% prediction interval (marked by the gray strip); The “x” represents the observed number of suicides outside the 95% prediction intervals. The solid line marks the predicted number of suicides in non-pandemic periods, whereas the dashed line represents the predicted number of suicides during the pandemic.
