# Supplementary material for: The Unusual Increase in Suicides Among Women in Japan During the COVID-19 Pandemic: A Time-series Analysis Until October 2021
Source: J Epidemiol. 2023 Jan 5;33(1):45–51. doi: 10.2188/jea.JE20220186 (PMC9727214; doi:10.2188/jea.JE20220186)
Supplement: Supplementary file 1 [file je-33-045-s001.pdf]

eTable 1. Estimated O/E ratio and its 95% confidence interval per job status, motive, and age category during Term I (March to December, 2020) as sensitivity analysis

	Estimation using unemployment rate in 2019			Estimation using unemployment rate in 2018		
	Total number of expectations	O/E ratio (95% CI)	*P*-value	Total number of expectations	O/E ratio (95% CI)	*P*-value
**Job status**						
Worker						
Employee	809.7	1.58 (1.50–1.67)	<0.001	816.9	1.57 (1.48–1.66)	<0.001
Self-employed	115.0	1.18 (0.99–1.39)	0.050	117.6	1.16 (0.97–1.36)	0.090
Non-worker						
Pension and unemployment insurance liver	1484.2	1.09 (1.04–1.15)	<0.001	1506.6	1.08 (1.03–1.13)	0.003
Housewife	749.1	1.32 (1.24–1.40)	<0.001	775.1	1.28 (1.20–1.36)	<0.001
Student	176.5	1.72 (1.54–1.92)	<0.001	175.8	1.73 (1.54–1.93)	<0.001
Unemployed	58.5	1.33 (1.06–1.65)	0.011	58.5	1.33 (1.06–1.65)	0.011
Other non-worker	994.0	1.33 (1.26–1.40)	<0.001	1020.5	1.30 (1.23–1.37)	<0.001
Unknown	478.0	0.40 (0.35–0.46)	<0.001	471.6	0.41 (0.35–0.47)	<0.001
**Motive**						
Health	3020.0	1.24 (1.20–1.28)	<0.001	3076.5	1.22 (1.18–1.26)	<0.001
Family	862.8	1.22 (1.15–1.29)	<0.001	869.5	1.21 (1.14–1.28)	<0.001
Economic	281.7	1.19 (1.07–1.33)	0.001	283.3	1.19 (1.07–1.32)	0.001
Work-related	172.9	1.61 (1.43–1.80)	<0.001	173.0	1.61 (1.43–1.80)	<0.001
Relationship	175.8	1.52 (1.35–1.71)	<0.001	178.4	1.50 (1.33–1.69)	<0.001
School-related	63.5	2.03 (1.70–2.40)	<0.001	63.2	2.04 (1.71–2.41)	<0.001
Other	208.5	1.64 (1.47–1.82)	<0.001	212.7	1.60 (1.44–1.78)	<0.001
Unknown reasons	1341.9	1.14 (1.08–1.20)	<0.001	1355.8	1.13 (1.07–1.19)	<0.001
**Age, years**						
<20	164.2	1.58 (1.40–1.78)	<0.001	162.2	1.60 (1.42–1.81)	<0.001
20–29	475.2	1.46 (1.36–1.57)	<0.001	478.7	1.45 (1.35–1.56)	<0.001
30–39	483.4	1.36 (1.26–1.47)	<0.001	499.4	1.32 (1.22–1.42)	<0.001
40–49	731.3	1.26 (1.19–1.35)	<0.001	743.0	1.24 (1.17–1.33)	<0.001
50–59	750.6	1.19 (1.11–1.27)	<0.001	756.8	1.18 (1.10–1.26)	<0.001
60–69	700.2	1.14 (1.06–1.22)	<0.001	719.5	1.11 (1.03–1.19)	0.003
70–79	814.8	1.14 (1.07–1.22)	<0.001	828.5	1.12 (1.05–1.20)	<0.001
≥80	724.1	1.05 (0.98–1.13)	0.159	733.0	1.04 (0.97–1.12)	0.285

CI, confidence interval; O/E ratio, ratio of observed to expected results.

eTable 2. Estimated O/E ratio and its 95% confidence interval per job status, motive, and age category during Term II (January to October, 2021) as sensitivity analysis

	Estimation using unemployment rate in 2019			Estimation using unemployment rate in 2018		
	Total number of expectations	O/E ratio (95% CI)	*P*-value	Total number of expectations	O/E ratio (95% CI)	*P*-value
**Job status**						
Worker						
Employee	812.0	1.51 (1.43–1.60)	<0.001	809.8	1.52 (1.43–1.60)	<0.001
Self-employed	117.6	1.10 (0.92–1.30)	0.295	116.5	1.10 (0.93–1.31)	0.249
Non-worker						
Pension and unemployment insurance liver	1496.3	1.08 (1.03–1.13)	0.003	1487.9	1.08 (1.03–1.14)	0.001
Housewife	757.8	1.20 (1.12–1.28)	<0.001	745.5	1.22 (1.14–1.30)	<0.001
Student	185.0	1.75 (1.56–1.94)	<0.001	185.5	1.74 (1.56–1.94)	<0.001
Unemployed	56.4	1.29 (1.02–1.61)	0.027	55.4	1.32 (1.04–1.64)	0.019
Other non-worker	991.0	1.31 (1.24–1.39)	<0.001	980.5	1.33 (1.26–1.40)	<0.001
Unknown	483.6	0.61 (0.54–0.68)	<0.001	489.9	0.60 (0.53–0.67)	<0.001
**Motive**						
Health	3031.2	1.15 (1.11–1.19)	<0.001	3010.6	1.16 (1.12–1.20)	<0.001
Family	860.6	1.27 (1.20–1.35)	<0.001	854.8	1.28 (1.21–1.36)	<0.001
Economic	282.6	1.31 (1.18–1.45)	<0.001	280.5	1.32 (1.19–1.46)	<0.001
Work-related	172.1	1.42 (1.25–1.60)	<0.001	171.7	1.42 (1.25–1.61)	<0.001
Relationship	177.9	1.56 (1.38–1.75)	<0.001	175.6	1.58 (1.40–1.77)	<0.001
School-related	65.2	1.43 (1.16–1.74)	<0.001	64.7	1.44 (1.17–1.75)	<0.001
Other	210.0	1.43 (1.28–1.60)	<0.001	207.9	1.45 (1.29–1.62)	<0.001
Unknown reasons	1350.0	1.16 (1.10–1.22)	<0.001	1345.8	1.16 (1.11–1.22)	<0.001
**Age, years**						
<20	168.0	1.57 (1.39–1.77)	<0.001	169.7	1.56 (1.38–1.75)	<0.001
20–29	476.1	1.62 (1.51–1.74)	<0.001	473.9	1.63 (1.51–1.74)	<0.001
30–39	497.8	1.25 (1.16–1.35)	<0.001	493.6	1.26 (1.17–1.36)	<0.001
40–49	736.8	1.22 (1.14–1.30)	<0.001	732.6	1.22 (1.14–1.30)	<0.001
50–59	737.4	1.23 (1.16–1.32)	<0.001	732.6	1.24 (1.16–1.32)	<0.001
60–69	715.2	1.01 (0.94–1.08)	0.799	707.0	1.02 (0.95–1.10)	0.572
70–79	819.3	1.14 (1.07–1.22)	<0.001	813.3	1.15 (1.08–1.23)	<0.001
≥80	720.9	1.03 (0.96–1.11)	0.370	716.1	1.04 (0.97–1.12)	0.280

CI, confidence interval; O/E ratio, ratio of observed to expected results.

Employee

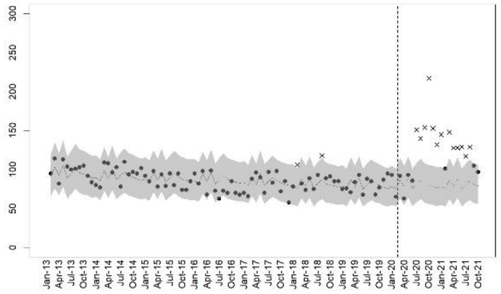

Self-employed

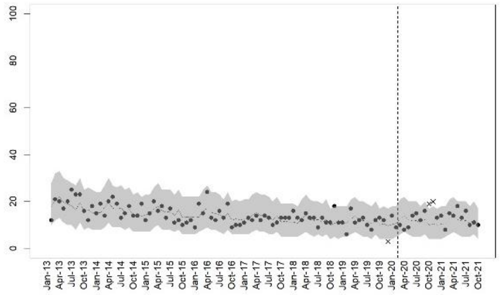

Pension/unemployment insurance liver

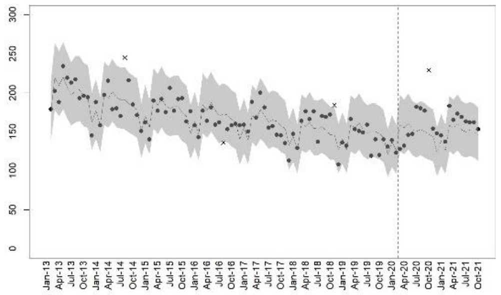

Housewife

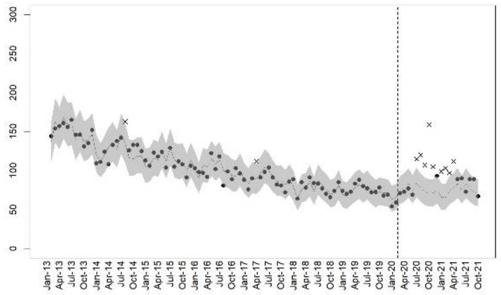

Student

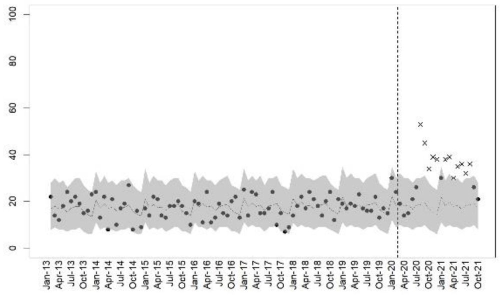

Unemployed

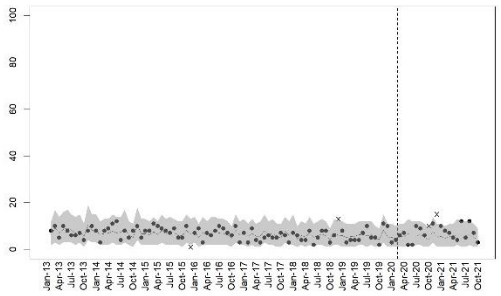

(Other) non-workers

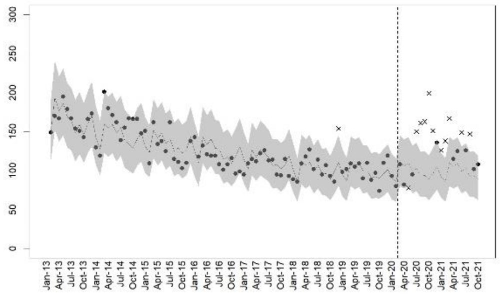

Unknown

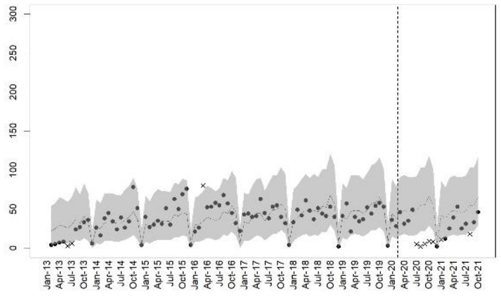

eFigure 1. Number of suicides among females by job status categories from January 2013 to October 2021. The dots represent the observed number of suicides within the 95% prediction interval (marked by the gray strip); The “x” represents the observed number of suicides outside the 95% prediction intervals. The solid line marks the predicted number of suicides in non-pandemic periods, whereas the dashed line represents the predicted number of suicides during the pandemic.

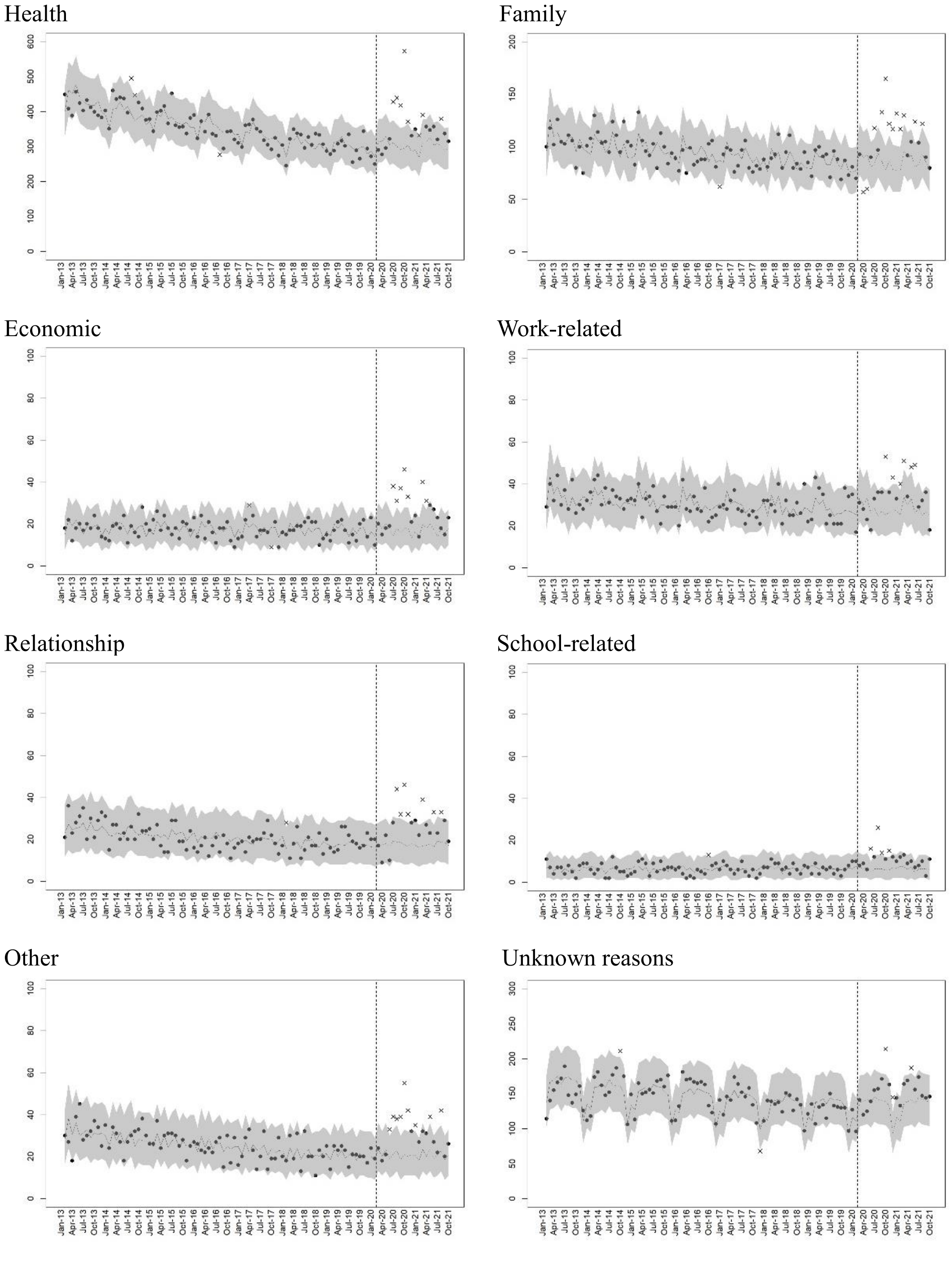

eFigure 2. Number of suicides among females by motive categories from January 2013 to October 2021. The dots represent the observed number of suicides within the 95% prediction interval (marked by the gray strip); The "x" represents the observed number of suicides outside the 95% prediction intervals. The solid line marks the predicted number of suicides in non-pandemic periods, whereas the dashed line represents the predicted number of suicides during the pandemic.

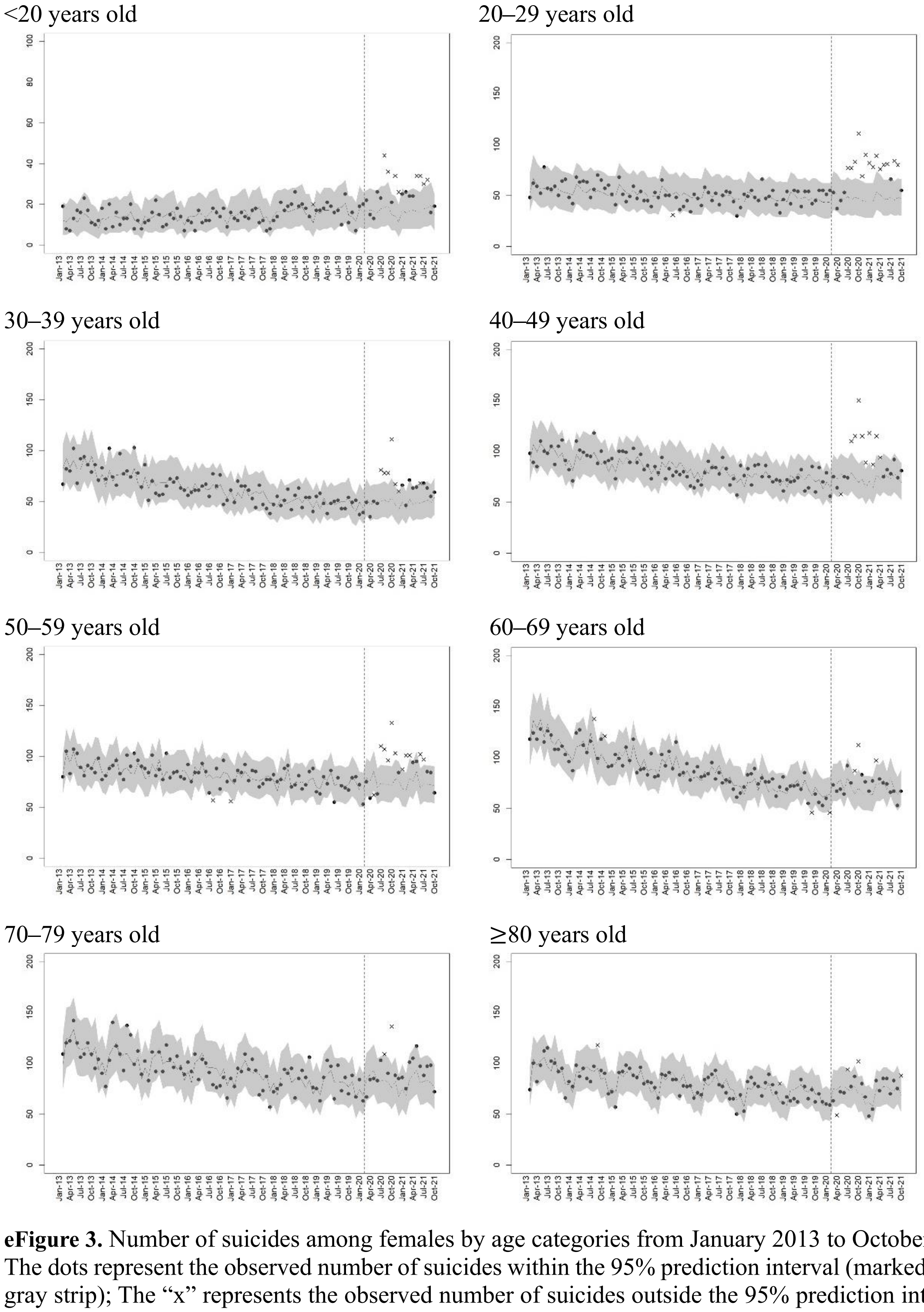

eFigure 3. Number of suicides among females by age categories from January 2013 to October 2021. The dots represent the observed number of suicides within the 95% prediction interval (marked by the gray strip); The “x” represents the observed number of suicides outside the 95% prediction intervals. The solid line marks the predicted number of suicides in non-pandemic periods, whereas the dashed line represents the predicted number of suicides during the pandemic.
